# Ureter as the Content in a Large Inguino Scrotal Hernia: A Diagnostic Dilemma for Unprepared Surgeons

**DOI:** 10.7759/cureus.45106

**Published:** 2023-09-12

**Authors:** Bal Jaskaran Singh, Abdus Samee, Arun Jain, Kasturi Sarathy

**Affiliations:** 1 General and Colorectal Surgery, Northern Care Alliance NHS Trust, Manchester, GBR; 2 Department of Surgery, Royal Oldham Hospital, Northern Care Alliance NHS Foundation Trust, Oldham, GBR; 3 Department of Urology, Manchester Foundation Trust, Manchester, GBR; 4 Department of Surgery, Northern Care Alliance NHS Trust, Manchester, GBR

**Keywords:** ureter, inguinal hernia repair, cm, copd, mdt, ct, gfr

## Abstract

We present a case of a 60-year-old gentleman, who was referred for an inguinal hernia repair. A previous CT scan had reported a right pelvic kidney and the presence of the right ureter in the hernial sac. He had no urinary symptoms and there was no biochemical evidence of compromised renal function. A renogram showed partial obstruction with an equally split function.

The findings were discussed in a urological multi-disciplinary team (MDT) meeting. A ureteric stent was advised to assist in the identification of the ureter during the surgery.

The patient had intraoperative stenting of the right ureter, followed by successful repair of the hernia. Six weeks later, the stent was removed, and the patient continues to remain well. Follow-up blood results showed normal renal functions.

## Introduction

Being a retroperitoneal structure, the presence of a ureter in an inguinal hernia is an extremely rare entity [1]. Spontaneous herniation of the native ureter or following renal transplant can result in the ureter dropping down into the hernial sac [2,3]. For an unprepared surgeon, this may pose a challenge while performing an inguinal hernia repair. Most of the cases of ureteric herniation are diagnosed during surgery or post-operatively. Patients with inguinal hernia along with unexplained obstructive uropathy should be evaluated properly before an elective surgery.

## Case presentation

A 60-year-old gentleman presented to our hospital with a large swelling in the right inguinal region extending into the scrotum for many years; it had become irreducible for the last few days. He had no urinary symptoms.

His past medical history included chronic obstructive pulmonary disease (COPD), morbid obesity and cholecystectomy. A previous computed tomography (CT) scan had reported the presence of the right pelvic kidney, as well as perinephric fat, along with the right ureter in the hernial sac reaching the scrotum and then looping back. The renogram showed partial obstruction with an equally split function.

Clinical examination confirmed a large incarcerated irreducible right inguinal hernia reaching up to the base of the right hemiscrotum. Abdominal and rectal examinations were normal. The baseline blood tests, including renal investigations, were within normal limits. The estimated glomerular filtration rate (GFR) was 77 ml/min/1.73 m^2^ (normal >90).

In view of the CT scan findings of the ureter as the content of the hernial sac, this patient was referred to a urologist for an assessment. The case was discussed in the urological multi-disciplinary team (MDT) meeting and ureteric stenting prior to scheduled surgery was advised.

**Figure 1:**
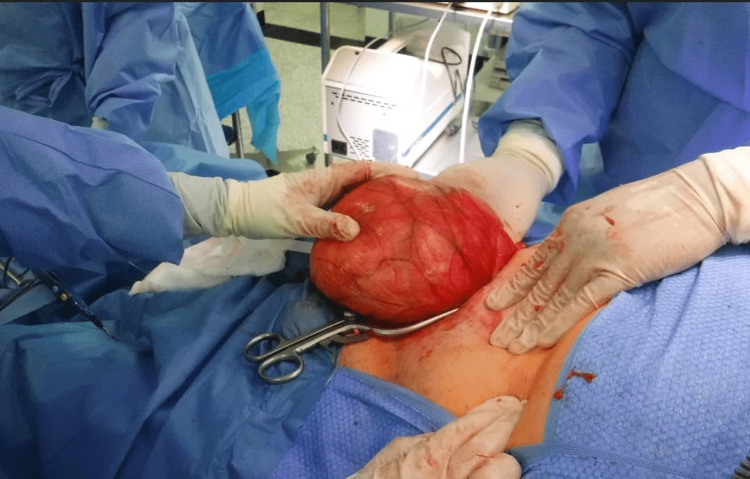
Intra-operative image of the hernial sac

**Figure 2:**
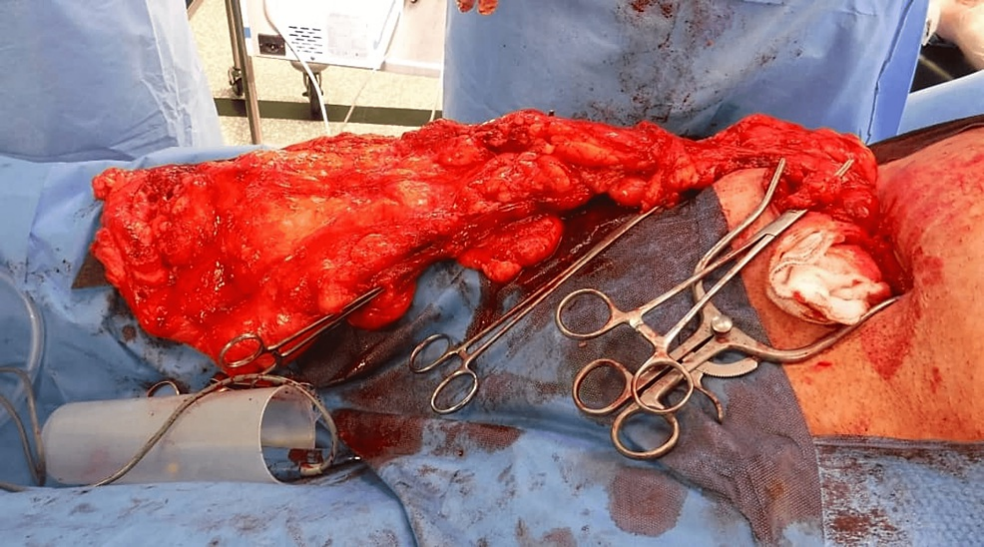
Opened hernial sac showing the incarcerated omentum

A preoperative ureteric stent insertion was unsuccessful and had to be taken out, as the stent fell back into the urinary bladder. The patient underwent intraoperative right ureteric stenting followed by elective hernia repair surgery. An inguinal crease incision was performed and an indirect hernia sac was dissected out (Figure 1). The opening of the hernial sac revealed a large incarcerated omental flap densely adherent to the right testes (Figure 2). The right ureter was found to be curled up in the hernia sac. It extended approximately 10 cm from the deep inguinal ring. The ureter was preserved throughout its length. Right orchidectomy (due to dense adhesions) along with omentectomy was performed, followed by mesh repair. Post-operatively, the patient had mild hematuria and hydronephrosis, but no urinary symptoms, and was managed conservatively. There was no evidence of injury to the ureter. The stent was removed after six weeks by the urologist.

## Discussion

Spontaneous herniation of the ureter in the hernial sac is an extremely rare event considering its retroperitoneal course. Less than 10 cases of ureteric hernia in native kidneys have been reported in the literature. Leroux, in 1880, reported the first case of herniation of the ureter in the inguinal canal during an autopsy [4]. Reichel reported the first intra-operative case, and Doumarshkin was the first to achieve a pre-operative diagnosis of ureteric herniation using intravenous pyelography in 1937 [5]. The ureter can not only herniate through the inguinal canal, but can also pass through the femoral ring, sciatic foramen, and diaphragm [6,7]. Men are more affected, typically after the fifth and sixth decades of life [8]. Most of these cases are symptomatic and diagnosed pre-operatively [9]. Unilateral hydronephrosis, urinary symptoms, or unexplained renal failure may be the presenting signs and symptoms, with most of the patients presenting with groin lump and renal failure [10].

Most cases of ureteric herniation occur in transplanted kidneys, due to the anterior location of the transplanted ureter in the space of Retzius. Herniation of the ureter occurs more commonly on the right side because, on the left side, the ureteric attachment is further reinforced in the retroperitoneum by the fascia of Toldt at the base of the sigmoid mesentery [11]. The proposed aetiology for this condition is the redundancy of the ureter, probably because of acquired nephroptosis while obesity is also considered a major risk factor [12].

In asymptomatic patients, this condition may be diagnosed during surgery, or later as a result of injury to the ureter that may or may not be identified during the surgery [12]. In diagnosed or suspected cases, a proper pre-operative assessment with imaging and renal function tests needs to be done to rule out any renal/obstructive pathology. The multi-disciplinary team approach, involving general surgeons and urologists, is the key to successful outcomes and minimising injury to the ureter and overall surgical morbidity.

The majority of cases of ureter in inguinal hernias have a large size and therefore have less chances of strangulation and obstructive uropathy. Even though rare, associated bladder herniation can lead to symptoms like frequency, dysuria, nocturia and hematuria [13]. Ureteral hernia when discovered incidentally requires further imaging of the renal tract to rule out any further anomaly or anatomical variation and to assess renal function.

Two anatomical variants of the ureter in inguinal hernia have been described, the more common being the paraperitoneal (80%) and the less common extraperitoneal (20%) type. The paraperitoneal type is proposed to be an acquired type. It occurs due to the adhesions between the posterior peritoneum and the ureter or because of the traction of underlying structures [14]. It might also contain a herniated bowel loop. It is recognised that the peritoneal sac is located lateral to the ureter, and there are more chances of obstruction when the ureter extends up to the scrotum [15].

The extraperitoneal type is believed to be a congenital defect resulting in the fusion of the ureter and genitoinguinal ligaments. It is thought to be due to the failure of separation of the ureteric bud from the Wolffian duct, both of which descend down to form the vas deferens and epididymis [15]. Therefore, the ureter herniates with retroperitoneal fat and without peritoneum in this type [16].

Computed tomography (CT) urogram and renal function tests provide information regarding the renal status and relevant anatomy. In the case of obstructive uropathy, the placement of a nephrostomy tube is advised to normalise renal function before surgery. In some cases, the surgeon opted for resection of the strangulated ureter followed by anastomosis or implantation into the bladder, due to the redundancy of the ureter [11,16]. In a few cases, simple hernia reduction and repair, taking extra precaution of the ureter, is sufficient.

Pre-operatively diagnosed cases can be planned and managed by peri-operative placement of a ureteral stent, which helps to identify the ureter, thus reducing the chances of ureteric injury [1].

Learning points

Considering the majority of the cases are asymptomatic the surgeons performing hernia surgery should be aware of this rare anomaly, especially in large inguinoscrotal hernias. Careful surgical dissection may avoid injury to the ureter and prevent significant patient morbidity.

Herniation of the ureter should be considered a possibility in any patient with unexplained unilateral hydronephrosis, renal dysfunction and urinary tract infection, and further imaging may be needed to rule out anatomical variation.

A multi-disciplinary team (MDT) approach involving a urologist to formulate a management plan would be in the best interest of the patient.

In diagnosed cases of ureteral hernia, ureteric stenting peri-operatively helps to identify and prevent injury to the ureter.

Joint surgical intervention, along with a urologist, especially in case of obstructive disease due to ureter herniation may improve outcome, particularly if ureteric resection followed by the implantation of a ureter is needed.

## Conclusions

Ureteral hernias have been reported in the literature, with most of them occurring in renal transplant patients, while they rarely occur in native kidneys. It is critical for a surgeon to have awareness of a potential ureteral herniation into the inguinal canal, in order to avoid potential injury to the ureter during surgery. A multi-disciplinary approach for a diagnosed case of ureteric hernia is required for the best outcome. Ureteric catheterisation or stent insertion is helpful in identifying the ureter during the hernia repair and preventing injury to it.
